# *k*-fractional integral trapezium-like inequalities through $(h,m)$-convex and $(\alpha,m)$-convex mappings

**DOI:** 10.1186/s13660-017-1586-6

**Published:** 2017-12-19

**Authors:** Hao Wang, Tingsong Du, Yao Zhang

**Affiliations:** Department of Mathematics, College of Science, China Three Gorges University, Yichang, 443002 China

**Keywords:** 26A33, 26A51, 26D07, 26D20, 41A55, $(h,m)$-convex functions, $(\alpha,m)$-convex functions, *k*-fractional integrals

## Abstract

In this paper, a new general identity for differentiable mappings via *k*-fractional integrals is derived. By using the concept of $(h,m)$-convexity, $(\alpha,m)$-convexity and the obtained equation, some new trapezium-like integral inequalities are established. The results presented provide extensions of those given in earlier works.

## Introduction

Let $f: I \subseteq\mathbb{R}\rightarrow\mathbb{R}$ be a convex mapping and $a, b \in I$ along with $a < b$. The inequality

1.1 $$ f\biggl(\frac{a+b}{2}\biggr)\leq\frac{1}{b-a} \int^{b}_{a}f(x)\,\mathrm {d}x\leq\frac{f(a)+f(b)}{2}, $$

named Hermite-Hadamard’s inequality, is one of the most famous results for convex mappings. This inequality (1.1) is also known as trapezium inequality.

The trapezium-type inequality has remained an area of great interest due to its wide applications in the field of mathematical analysis. Many researchers generalized and extended it via mappings of different classes. For recent results, for example, see [1–7] and the references mentioned in these papers.

In 2013, Sarikaya et al. [8] established the following theorem by utilizing Riemann-Liouville fractional integrals.

### Theorem 1.1

*Let* $f:[a,b]\rightarrow\mathbb{R}$ *be a positive function along with* $0\leq a< b$, *and let* $f\in L^{1}[a,b]$. *Suppose that* *f* *is a convex function on* $[a,b]$, *then the following inequalities for fractional integrals hold*:

1.2 $$ f\biggl(\frac{a+b}{2}\biggr) \leq\frac{\Gamma(\mu+1)}{2(b-a)^{\mu}} \bigl[J^{\mu }_{a^{+}}f(b)+J^{\mu}_{b^{-}}f(a) \bigr]\leq\frac{f(a)+f(b)}{2}, $$

*where the symbols* $J^{\mu}_{a^{+}} f$ *and* $J^{\mu}_{b^{-}} f$ *denote respectively the left*-*sided and right*-*sided Riemann*-*Liouville fractional integrals of order* $\mu>0$ *defined by*

$$ J^{\mu}_{a^{+}}f(x) = \frac{1}{\Gamma(\mu)} \int^{x}_{a}(x-t)^{\mu -1}f(t)\,\mathrm{d}t,\quad a< x $$

*and*

$$ J^{\mu}_{b^{-}}f(x) = \frac{1}{\Gamma(\mu)} \int^{b}_{x}(t-x)^{\mu -1}f(t)\,\mathrm{d}t,\quad x< b. $$

*Here*, $\Gamma(\mu)$ *is the gamma function and its definition is* $\Gamma(\mu)=\int_{0}^{\infty}e^{-t}t^{\mu-1}\,\mathrm{d}t$. *It is to be noted that* $J^{0}_{a^{+}}f(x)=J^{0}_{b^{-}}f(x)=f(x)$.

In the case of $\mu=1$, the fractional integral recaptures the classical integral.

Because of the extensive application of Riemann-Liouville fractional integrals, some authors extended their studies to fractional trapezium-type inequalities via mappings of different classes. For example, refer to [9–12] for convex mappings, to [13] for *s*-convex mappings, to [14] for $(s,m)$-convex mappings, to [15] for *r*-convex mappings, to [16] for harmonically convex mappings, to [17] for *s*-Godunova-Levin mappings, to [18, 19] for preinvex mappings, to [20] for MT_*m*_-preinvex mappings, to [21] for *h*-convex mappings and to references cited therein.

In [22], Mubeen and Habibullah introduced the following class of fractional derivatives.

### Definition 1.1

([22])

Let $f\in L^{1}[a,b]$, then *k*-Riemann-Liouville fractional derivatives ${}_{k}J^{\mu}_{a^{+}}f(x)$ and ${}_{k}J^{\mu}_{b^{-}}f(x)$ of order $\mu>0$ are given as

$$ _{k}J^{\mu}_{a^{+}}f(x) = \frac{1}{k\Gamma_{k}(\mu)} \int^{x}_{a}(x-t)^{\frac {\mu}{k}-1}f(t)\,\mathrm{d}t\quad (0\leq a< x< b) $$

and

$$ _{k}J^{\mu}_{b^{-}}f(x) = \frac{1}{k\Gamma_{k}(\mu)} \int^{b}_{x}(t-x)^{\frac {\mu}{k}-1}f(t)\,\mathrm{d}t \quad (0 \leq a< x< b), $$

respectively, where $k>0$ and $\Gamma_{k}(\mu)$ is the *k*-gamma function defined by $\Gamma_{k}(\mu)=\int^{\infty}_{0} t^{\mu-1}\times e^{-\frac{t^{k}}{k}}\,\mathrm{d}t$. Furthermore, $\Gamma {}_{k}(\mu+k)=\mu\Gamma{}_{k}(\mu)$ and ${}_{k}J^{0}_{a^{+}}f(x)={}_{k}J^{0}_{b^{-}}f(x)=f(x)$.

The concept of *k*-Riemann-Liouville fractional integral is an important extension of Riemann-Liouville fractional integrals. We want to stress here that for $k\neq1$ the properties of *k*-Riemann-Liouville fractional integrals are quite dissimilar from those of general Riemann-Liouville fractional integrals. For this, the *k*-Riemann-Liouville fractional integrals have aroused the interest of many researchers. Properties concerning this operator can be sought out [23–26], and for the bounds for integral inequality related to this operator, the reader can refer to [27–29] and the references mentioned in these papers.

Motivated and inspired by the recent research in this field, we obtain some *k*-Riemann-Liouville fractional integral of trapezium-type inequalities for $(h,m)$-convex mappings and $(\alpha,m)$-convex mappings. The results presented in this paper provide extensions of those given in earlier works.

To end this section, we restate some special functions and definitions.

1. The beta function:
  $$ \beta(x,y)= \int^{1}_{0}t^{x-1}(1-t)^{y-1} \,\mathrm{d}t=\frac{\Gamma (x)\Gamma(y)}{\Gamma(x+y)}, \quad\forall x,y>0. $$
2. The incomplete beta function:
  $$ \beta(a,x,y)= \int^{a}_{0}t^{x-1}(1-t)^{y-1} \,\mathrm{d}t,\quad 0< a< 1, x,y>0. $$

### Definition 1.2

([30])

The function $f:[0,b]\rightarrow\mathbb{R}$ is named $(\alpha,m)$-convex if, for every $x,y\in[0,b]$ and $t\in[0,1]$, the following inequality holds:

$$ f\bigl(tx+m(1-t)y\bigr)\leq t^{\alpha}f(x)+m \bigl(1-t^{\alpha}\bigr)f(y), $$

where $(\alpha,m)\in(0,1]\times(0,1]$.

### Definition 1.3

([31])

The function $f:[0,b]\rightarrow\mathbb{R}$ is called *m*-MT-convex if *f* is non-negative and, for all $x, y\in[0,b]$ and $t\in(0, 1)$, with $m\in(0,1]$, it satisfies the following inequality:

$$ f\bigl(tx+m(1-t)y\bigr)\leq\frac{\sqrt{t}}{2\sqrt{1-t}}f(x)+ \frac{m\sqrt {1-t}}{2\sqrt{t}}f(y). $$

### Definition 1.4

([32])

Let $h: (0,1)\subseteq J\rightarrow\mathbb{R}$ be a non-negative function. A function $f:I\rightarrow\mathbb{R}$ is said to be *h*-convex if *f* is non-negative and

$$ f\bigl(tx+(1-t)y\bigr)\leq h(t)f(x)+h(1-t)f(y) $$

holds for all $x,y\in I$ and $t\in[0,1]$.

### Definition 1.5

([33])

Let $f:I\subseteq\mathbb{R}\rightarrow\mathbb{R}$ be a non-negative function. A function $f:I\rightarrow\mathbb{R}$ is said to be $tgs$-convex if the inequality

$$ f\bigl(tx+ (1-t)y\bigr)\leq t(1-t)\bigl[f(x)+f(y)\bigr] $$

holds for all $x, y \in I $ and $t\in(0,1)$.

### Definition 1.6

([34])

Let $h: (0,1)\subseteq J\rightarrow\mathbb{R}$ be a non-negative function. A function $f:[0,b]\rightarrow\mathbb{R}$ is named $(h,m)$-convex if *f* is non-negative and

$$ f\bigl(tx+m(1-t)y\bigr)\leq h(t)f(x)+mh(1-t)f(y) $$

holds for all $x,y\in[0,b]$, $t\in(0,1)$ and some fixed $m\in(0,1]$.

Clearly, when putting $h(t)=t(1-t)$ in Definition 1.6, *f* becomes an $(m,tgs)$-convex function on $[0,b]$ as follows.

### Definition 1.7

The function $f:[0,b]\rightarrow\mathbb{R}$ is named $(m,tgs)$-convex if *f* is non-negative and

$$ f\bigl(tx+m(1-t)y\bigr)\leq t(1-t)\bigl[f(x)+mf(y)\bigr] $$

holds for all $x,y\in[0,b]$, $t\in(0,1)$ and some fixed $m\in(0,1]$.

Note that, if we choose $m=1$ in Definition 1.7, *f* reduces to a $tgs$-convex function in Definition 1.5.

## A lemma

To prove our main results, we consider the following new lemma.

### Lemma 2.1

*Let* $f:I\subseteq\mathbb{R}\rightarrow\mathbb{R}$ *be a differentiable mapping on* $I^{o}$ (*the interior of* *I*) *with* $0\leq a< mr$, $a,r\in I$, *for some fixed* $m \in(0,1]$. *If* $f'\in L^{1}[a,mr]$, *then the following equality for* *k*-*fractional integral along with* $\lambda\in(0,1]\backslash\frac{1}{2}$, $k>0$ *and* $\mu>0$ *exists*:

2.1 $$\begin{aligned} &\mathcal{T}_{k,\mu}(m,\lambda,r) \\ &\quad = \int^{1}_{0}\bigl((1-t)^{\frac{\mu}{k}}-t^{\frac{\mu}{k}}\bigr) f'\biggl(t\bigl(\lambda a+m(1- \lambda)r\bigr)+m(1-t) \biggl(\lambda r+(1-\lambda)\frac{a}{m}\biggr) \biggr)\,\mathrm{d}t, \end{aligned}$$

*where*

2.2 $$\begin{aligned} &\mathcal{T}_{k,\mu}(m,\lambda,r) \\ &\quad:=-\frac{f(m\lambda r+(1-\lambda)a)+f(\lambda a+m(1-\lambda)r)}{(1-2\lambda)(mr-a)}+\frac{\Gamma_{k}(\mu+k)}{ (1-2\lambda)^{\frac{\mu}{k}+1}(mr-a)^{\frac{\mu}{k}+1}} \\ &\qquad{} \times\bigl[{}_{k}J^{\mu}_{(m\lambda r+(1-\lambda)a)^{+}}f\bigl(\lambda a+m(1-\lambda)r\bigr) +{}_{k}J^{\mu}_{(\lambda a+m(1-\lambda)r)^{-}}f\bigl(m \lambda r+(1-\lambda)a\bigr)\bigr]. \end{aligned}$$

### Proof

It suffices to note that

2.3 $$\begin{aligned} I^{*}={}& \int^{1}_{0}\bigl((1-t)^{\frac{\mu}{k}}-t^{\frac{\mu}{k}}\bigr)f' \biggl(t\bigl(\lambda a+m(1- \lambda)r\bigr)+m(1-t) \biggl(\lambda r+(1-\lambda )\frac{a}{m}\biggr) \biggr)\,\mathrm{d}t \\ ={}&\biggl[ \int^{1}_{0}(1-t)^{\frac{\mu}{k}} f'\biggl(t\bigl(\lambda a+m(1-\lambda)r\bigr)+m(1-t) \biggl(\lambda r+(1-\lambda)\frac{a}{m}\biggr)\biggr)\,\mathrm{d}t\biggr] \\ &{} +\biggl[- \int^{1}_{0}t^{\frac{\mu}{k}} f'\biggl(t\bigl(\lambda a+m(1-\lambda)r\bigr)+m(1-t) \biggl(\lambda r+(1-\lambda)\frac{a}{m}\biggr)\biggr)\,\mathrm{d}t\biggr] \\ :={}&I_{1}+I_{2}. \end{aligned}$$

Integrating by parts, we get

2.4 $$\begin{aligned} I_{1}={}&\biggl[ \int^{1}_{0}(1-t)^{\frac{\mu}{k}} f'\biggl(t\bigl(\lambda a+m(1-\lambda)r\bigr)+m(1-t) \biggl(\lambda r+(1-\lambda)\frac{a}{m}\biggr)\biggr)\,\mathrm{d}t\biggr] \\ ={}&\frac{f(t(\lambda a+m(1-\lambda)r)+m(1-t)(\lambda r+(1-\lambda)\frac{a}{m}))(1-t)^{\frac{\mu}{k}}}{ (1-2\lambda)(mr-a)}\Big|_{0}^{1} \\ &{} +\frac{\frac{\mu}{k}}{(1-2\lambda)(mr-a)}\biggl[ \int ^{1}_{0}(1-t)^{\frac{\mu}{k}-1} f\biggl(t\bigl( \lambda a+m(1-\lambda)r\bigr) \\ &{} +m(1-t) \biggl(\lambda r +(1-\lambda)\frac{a}{m}\biggr)\biggr)\,\mathrm {d}t\biggr] \\ ={}&{-}\frac{f(m\lambda r+(1-\lambda)a)}{(1-2\lambda)(mr-a)} +\frac {\frac{\mu}{k}}{(1-2\lambda)(mr-a)} \\ &{} \times\biggl[ \int^{1}_{0}(1-t)^{\frac{\mu}{k}-1} f\biggl(t\bigl( \lambda a+m(1-\lambda)r\bigr)+m(1-t) \biggl(\lambda r+(1-\lambda) \frac{a}{m}\biggr)\biggr)\,\mathrm{d}t\biggr]. \end{aligned}$$

Let $x=t(\lambda a+m(1-\lambda)r)+m(1-t)(\lambda r+(1-\lambda)\frac{a}{m})$, $t\in[0,1]$, equality (2.4) can be written as

2.5 $$\begin{aligned} I_{1}={}&{-}\frac{f(m\lambda r+(1-\lambda)a)}{(1-2\lambda)(mr-a)} \\ & {}+\frac{\frac{\mu}{k}}{(1-2\lambda)^{\frac{\mu}{k}}(mr-a)^{\frac{\mu }{k}+1}} \int^{\lambda a+m(1-\lambda)r}_{m\lambda r+(1-\lambda)a}\bigl(\lambda a+m(1-\lambda )r-x \bigr)^{\frac{\mu}{k}-1}f(x)\,\mathrm{d}x \\ ={}&{-}\frac{f(m\lambda r+(1-\lambda)a)}{(1-2\lambda)(mr-a)} \\ &{} +\frac{\Gamma_{k}(\mu+k)}{ (1-2\lambda)^{\frac{\mu}{k}+1}(mr-a)^{\frac{\mu}{k}+1}}{}_{k}J^{\mu }_{(m\lambda r+(1-\lambda)a)^{+}}f\bigl( \lambda a+m(1-\lambda)r\bigr), \end{aligned}$$

and similarly we get

2.6 $$\begin{aligned} I_{2}={}& \int^{1}_{0}t^{\frac{\mu}{k}} f'\biggl(t\bigl(\lambda a+m(1-\lambda)r\bigr)+m(1-t) \biggl(\lambda r+(1-\lambda)\frac{a}{m}\biggr)\biggr)\,\mathrm{d}t \\ ={}&{-}\frac{t^{\frac{\mu}{k}}f(t(\lambda a+m(1-\lambda)r )+m(1-t)(\lambda r+(1-\lambda)\frac{a}{m}))}{(1-2\lambda)(mr-a)}\Big|_{0}^{1} \\ &{} + \frac{\frac{\mu}{k}}{(1-2\lambda)(mr-a)} \\ &{}\times\int^{1}_{0} t^{\frac{\mu}{k}-1}f\biggl(t\bigl(\lambda a+m(1-\lambda)r\bigr)+m(1-t) \biggl(\lambda r+(1-\lambda)\frac{a}{m} \biggr)\biggr)\,\mathrm{d}t \\ ={}&{-}\frac{f(\lambda a+m(1-\lambda)r)}{(1-2\lambda)(mr-a)} \\ & {}+ \frac{\Gamma_{k}(\mu+k)}{ (1-2\lambda)^{\frac{\mu}{k}+1}(mr-a)^{\frac{\mu}{k}+1}}{}_{k}J^{\mu }_{(\lambda a+m(1-\lambda)r)^{-}}f\bigl(m \lambda r+(1-\lambda)a\bigr). \end{aligned}$$

Hence, using (2.5) and (2.6) in (2.3), we can obtain the desired result. □

### Corollary 2.1

*In Lemma* 2.1, *for* $k=1$, *we can get the result for Riemann*-*Liouville fractional integral*.

### Corollary 2.2

*In Lemma* 2.1, *if we put* $\lambda=0$, *we get*

2.7 $$\begin{aligned} &{-}\frac{f(a)+f(mr)}{mr-a}+\frac{\Gamma_{k}(\mu+k)}{(mr-a)^{\frac{\mu }{k}+1}} \bigl[{}_{k}J^{\mu}_{a^{+}}f(mr)+{}_{k}J^{\mu}_{mr^{-}}f(a) \bigr] \\ &\quad= \int^{1}_{0}\bigl((1-t)^{\frac{\mu}{k}}-t^{\frac{\mu}{k}}\bigr)f' \bigl(tmr+(1-t)a\bigr) \,\mathrm{d}t. \end{aligned}$$

*Similarly*, *taking* $\lambda=1$ *in Lemma* 2.1, *we obtain*

2.8 $$\begin{aligned} &\frac{f(a)+f(mr)}{mr-a}+\frac{\Gamma_{k}(\mu+k)}{(-1)^{\frac{\mu }{k}+1}(mr-a)^{\frac{\mu}{k}+1}} \bigl[{}_{k}J^{\mu }_{mr^{+}}f(a)+{}_{k}J^{\mu}_{a^{-}}f(mr) \bigr] \\ &\quad= \int^{1}_{0}\bigl((1-t)^{\frac{\mu}{k}}-t^{\frac{\mu}{k}}\bigr)f' \bigl(ta+(1-t)mr\bigr) \,\mathrm{d}t. \end{aligned}$$

*Note that* ${}_{k}J^{\mu}_{mr^{+}}f(a)+{}_{k}J^{\mu}_{a^{-}}f(mr) =(-1)^{\frac{\mu}{k}}[{}_{k}J^{\mu}_{a^{+}}f(mr)+{}_{k}J^{\mu }_{mr^{-}}f(a)]$, *it is easy to see that identity* (2.8) *is equal to identity* (2.7).

### Remark 2.1

(i) In Corollary 2.1, if we put $r=b$, then one can obtain Lemma 3.1 which is proved in [35]. Further, if we take $m=1$, then we obtain Lemma 2.1 in [12].
(ii) In Corollary 2.2,
  - if we put $k=1=m$, then we obtain Lemma 3 in [11],
  - if we put $k=1=m$ and $r=b$, then we obtain Lemma 2 in [8],
  - if we put $k=m=\mu=1$ and $r=b$, then we obtain Lemma 2.1 in [36].

## *k*-fractional integral inequalities for $(h,m)$-convex functions

In what follows, we establish some *k*-fractional integral inequalities for $(h,m)$-convex functions by using Lemma 2.1.

### Theorem 3.1

*Let* $h:J\subseteq\mathbb{R}\rightarrow\mathbb{R}\ ([0,1]\subseteq J)$ *be a non*-*negative function*, *and let* $f:I\subseteq\mathbb{R}\rightarrow\mathbb{R}$ *be a differentiable mapping on* $I^{o}$ *along with* $a,r\in I$, $0\leq a < mr$, *for some fixed* $m\in(0,1]$. *If* $f'\in L^{1}[a,mr]$ *and* $\vert f' \vert ^{q}$ *for* $q\geq1$ *is* $(h,m)$-*convex on* $[a,mr]$, *then the following inequality exists*:

3.1 $$\begin{aligned} \bigl\vert \mathcal{T}_{k,\mu}(m,\lambda,r) \bigr\vert \leq{}&\biggl[\frac{2k}{\mu+k}\biggl(1-\frac{1}{2^{\frac{\mu}{k}}}\biggr) \biggr]^{1-\frac{1}{q}} \biggl[ \int^{\frac{1}{2}}_{0}\bigl((1-t)^{\frac{\mu}{k}}-t^{\frac{\mu }{k}}\bigr) \bigl(h(t)+h(1-t)\bigr)\,\mathrm{d}t \biggr]^{\frac{1}{q}} \\ & {}\times\biggl[ \bigl\vert f'\bigl(\lambda a+m(1-\lambda)r\bigr) \bigr\vert ^{q}+m \biggl\vert f'\biggl(\lambda r+(1- \lambda)\frac{a}{m}\biggr) \biggr\vert ^{q} \biggr]^{\frac{1}{q}}, \end{aligned}$$

*where* $\lambda\in(0,1]\backslash\frac{1}{2}$, $k>0$ *and* $\mu>0$.

### Proof

Case 1: $q=1$. Applying Lemma 2.1 and the $(h,m)$-convexity of $\vert f' \vert $, we have

$$\begin{aligned} & \bigl\vert \mathcal{T}_{k,\mu}(m,\lambda,r) \bigr\vert \\ &\quad= \biggl\vert \int^{1}_{0}\bigl((1-t)^{\frac{\mu}{k}}-t^{\frac{\mu}{k}}\bigr) f'\biggl(t\bigl(\lambda a+m(1- \lambda)r\bigr)+m(1-t) \biggl(\lambda r+(1-\lambda)\frac{a}{m}\biggr) \biggr)\,\mathrm{d}t \biggr\vert \\ &\quad\leq \int^{1}_{0} \bigl\vert (1-t)^{\frac{\mu}{k}}-t^{\frac{\mu}{k}} \bigr\vert \biggl\vert f'\biggl(t \bigl(\lambda a+m(1-\lambda)r\bigr)+m(1-t) \biggl(\lambda r+(1-\lambda) \frac{a}{m}\biggr)\biggr) \biggr\vert \,\mathrm{d}t \\ &\quad\leq \int^{1}_{0} \bigl\vert (1-t)^{\frac{\mu}{k}}-t^{\frac{\mu}{k}} \bigr\vert \\ &\qquad{} \times\biggl[h(t) \bigl\vert f'\bigl(\lambda a+m(1-\lambda)r \bigr) \bigr\vert +mh(1-t) \biggl\vert f'\biggl(\lambda r+(1- \lambda)\frac{a}{m}\biggr) \biggr\vert \biggr]\,\mathrm{d}t \\ &\quad= \int^{\frac{1}{2}}_{0}\bigl((1-t)^{\frac{\mu}{k}}-t^{\frac{\mu}{k}}\bigr) \\ &\qquad{} \times\biggl[h(t) \bigl\vert f'\bigl(\lambda a+m(1-\lambda)r \bigr) \bigr\vert +mh(1-t) \biggl\vert f'\biggl(\lambda r+(1- \lambda)\frac{a}{m}\biggr) \biggr\vert \biggr]\,\mathrm{d}t \\ &\qquad{} + \int^{1}_{\frac{1}{2}}\bigl(t^{\frac{\mu}{k}}-(1-t)^{\frac{\mu}{k}} \bigr) \\ &\qquad{} \times\biggl[h(t) \bigl\vert f'\bigl(\lambda a+m(1-\lambda)r \bigr) \bigr\vert +mh(1-t) \biggl\vert f'\biggl(\lambda r+(1- \lambda)\frac{a}{m}\biggr) \biggr\vert \biggr]\,\mathrm{d}t, \end{aligned}$$

where we use the fact that

$$\begin{aligned} & \int^{1}_{\frac{1}{2}}t^{\frac{\mu}{k}}h(t) \,\mathrm{d}t= \int^{\frac {1}{2}}_{0}(1-t)^{\frac{\mu}{k}}h(1-t) \,\mathrm{d}t, \\ & \int^{1}_{\frac{1}{2}}t^{\frac{\mu}{k}}h(1-t) \,\mathrm{d}t= \int^{\frac{1}{2}}_{0}(1-t)^{\frac{\mu}{k}}h(t) \,\mathrm{d}t, \\ & \int^{1}_{\frac{1}{2}}(1-t)^{\frac{\mu}{k}}h(t) \,\mathrm{d}t= \int^{\frac {1}{2}}_{0}t^{\frac{\mu}{k}}h(1-t) \,\mathrm{d}t \end{aligned}$$

and

$$ \int^{1}_{\frac{1}{2}}(1-t)^{\frac{\mu}{k}}h(1-t) \,\mathrm{d}t= \int^{\frac {1}{2}}_{0}t^{\frac{\mu}{k}}h(t) \,\mathrm{d}t. $$

By calculation,

$$\begin{aligned} & \int^{1}_{0} \bigl\vert (1-t)^{\frac{\mu}{k}}-t^{\frac{\mu}{k}} \bigr\vert \biggl[h(t) \bigl\vert f'\bigl(\lambda a+m(1-\lambda)r\bigr) \bigr\vert +mh(t) \biggl\vert f'\biggl(\lambda r+(1-\lambda)\frac{a}{m}\biggr) \biggr\vert \biggr]\,\mathrm{d}t \\ &\quad\leq\biggl[ \int^{\frac{1}{2}}_{0}\bigl((1-t)^{\frac{\mu}{k}}-t^{\frac {\mu}{k}} \bigr) \bigl(h(t)+h(1-t)\bigr)\,\mathrm{d}t\biggr] \\ & \qquad{}\times\biggl[ \bigl\vert f'\bigl(\lambda a +m(1-\lambda)r \bigr) \bigr\vert +m \biggl\vert f' \biggl(\lambda r+(1-\lambda) \frac{a}{m}\biggr) \biggr\vert \biggr]. \end{aligned}$$

Case 2: $q>1$. Employing Lemma 2.1, the power mean inequality and the $(h,m)$-convexity of $\vert f' \vert ^{q}$ leads to

$$\begin{aligned} & \int^{1}_{0} \bigl\vert (1-t)^{\frac{\mu}{k}}-t^{\frac{\mu}{k}} \bigr\vert \biggl\vert f'\biggl(t \bigl(\lambda a+m(1-\lambda)r\bigr)+m(1-t) \biggl(\lambda r+(1-\lambda) \frac{a}{m}\biggr)\biggr) \biggr\vert \,\mathrm{d}t \\ &\quad\leq\biggl[ \int^{1}_{0} \bigl\vert (1-t)^{\frac{\mu}{k}}-t^{\frac{\mu}{k}} \bigr\vert \,\mathrm{d}t\biggr]^{1-\frac{1}{q}} \\ &\qquad{} \times\biggl[ \int^{1}_{0} \bigl\vert (1-t)^{\frac{\mu}{k}}-t^{\frac{\mu}{k}} \bigr\vert \biggl\vert f'\biggl(t \bigl(\lambda a+m(1-\lambda)r\bigr)+m(1-t) \biggl(\lambda r+(1-\lambda) \frac{a}{m}\biggr)\biggr) \biggr\vert ^{q}\,\mathrm{d}t \biggr]^{\frac {1}{q}} \\ &\quad\leq\biggl[ \int^{\frac{1}{2}}_{0}\bigl((1-t)^{\frac{\mu}{k}}-t^{\frac{\mu }{k}}\bigr)\,\mathrm{d}t+ \int^{1}_{\frac{1}{2}}\bigl(t^{\frac{\mu }{k}}-(1-t)^{\frac{\mu}{k}}\bigr)\,\mathrm{d}t\biggr]^{1-\frac{1}{q}} \\ & \qquad{}\times\biggl\{ \int^{1}_{0} \bigl\vert (1-t)^{\frac{\mu}{k}}-t^{\frac{\mu}{k}} \bigr\vert \biggl[h(t) \bigl\vert f'\bigl(\lambda a+m(1-\lambda)r\bigr) \bigr\vert ^{q} \\ &\qquad{} +mh(1-t) \biggl\vert f'\biggl(\lambda r+(1-\lambda) \frac{a}{m}\biggr) \biggr\vert ^{q} \biggr]\,\mathrm{d}t\biggr\} ^{\frac{1}{q}} \\ &\quad=\biggl[\frac{2k}{\mu+k}\biggl(1-\frac{1}{2^{\frac{\mu}{k}}}\biggr) \biggr]^{1-\frac{1}{q}} \biggl[ \int^{\frac{1}{2}}_{0}\bigl((1-t)^{\frac{\mu}{k}}-t^{\frac{\mu }{k}}\bigr) \bigl(h(t)+h(1-t)\bigr)\,\mathrm{d}t \biggr]^{\frac{1}{q}} \\ &\qquad{} \times\biggl[ \bigl\vert f'\bigl(\lambda a+m(1-\lambda)r\bigr) \bigr\vert ^{q}+m \biggl\vert f'\biggl(\lambda r+(1- \lambda)\frac{a}{m}\biggr) \biggr\vert ^{q} \biggr]^{\frac{1}{q}}. \end{aligned}$$

This completes the proof. □

Now, we point out some special cases of Theorem 3.1.

### Corollary 3.1

*In Theorem* 3.1, *if we choose* $h(t)=t$ *and* $r=b$, *then we derive the following inequality for* *m*-*convex functions*:

3.2 $$\begin{aligned} & \bigl\vert \mathcal{T}_{k,\mu}(m,\lambda,b) \bigr\vert \\ &\quad\leq\frac{2k}{\mu+k}\biggl[1-\frac{1}{2^{\frac{\mu}{k}}}\biggr] \biggl[ \frac { \vert f'(\lambda a+m(1-\lambda)b) \vert ^{q}+m \vert f'(\lambda b+(1-\lambda)\frac{a}{m}) \vert ^{q}}{2}\biggr]^{\frac{1}{q}}. \end{aligned}$$

*Especially if we put* $k=1$, *we obtain Theorem *3.2 *in* [35].

### Corollary 3.2

*In Theorem* 3.1, *if we choose* $h(t)=t$, $m=1$ *and* $\lambda=0$ *or* $\lambda=1$, *then we derive the following inequality for convex functions*:

$$\begin{aligned} & \biggl\vert -\frac{f(a)+f(r)}{r-a}+\frac{\Gamma_{k}(\mu+k)}{(r-a)^{\frac{\mu }{k}+1}} \bigl[{}_{k}J^{\mu}_{a^{+}}f(r)+{}_{k}J^{\mu}_{r^{-}}f(a) \bigr] \biggr\vert \\ &\quad \leq\frac{2k}{\mu+k}\biggl[1-\frac{1}{2^{\frac{\mu}{k}}}\biggr] \biggl[ \frac { \vert f'(r) \vert ^{q}+ \vert f'(a) \vert ^{q}}{2}\biggr]^{\frac{1}{q}}. \end{aligned}$$

### Remark 3.1

In Corollary 3.2,

- if we put $k=1$, we can obtain Theorem 2.3 in [12],
- if we put $k=1$ and $r=b$, we can obtain Corollary 2.4 in [12],
- if we put $k=1=\mu$ and $r=b$, we can obtain Theorem 1 in [37],
- if we put $\mu=q=k=1$ and $r=b$, we can obtain Theorem 2.2 in [36].

### Corollary 3.3

*In Theorem* 3.1, *if we choose* $h(t)=t^{s}$, $s\in(0,1]$, *then we have the following inequality for* $(s,m)$-*Breckner convex functions*:

3.3 $$\begin{aligned} \bigl\vert \mathcal{T}_{k,\mu}(m,\lambda,r) \bigr\vert \leq{}&\biggl[\frac{2k}{\mu+k}\biggl(1-\frac{1}{2^{\frac{\mu}{k}}}\biggr) \biggr]^{1-\frac{1}{q}} \\ & {}\times\biggl[\beta\biggl(\frac{1}{2}, s+1, \frac{\mu}{k}+1\biggr)- \beta \biggl(\frac{1}{2}, \frac{\mu}{k}+1, s+1\biggr) \\ &{} +\frac{k}{k(s+1)+\mu}-\frac{k}{k(s+1)+\mu}\biggl(\frac{1}{2} \biggr)^{\frac {sk+\mu}{k}}\biggr]^{\frac{1}{q}} \\ & {}\times\biggl[ \bigl\vert f'\bigl(\lambda a+m(1-\lambda)r\bigr) \bigr\vert ^{q}+m \biggl\vert f'\biggl(\lambda r+(1- \lambda)\frac{a}{m}\biggr) \biggr\vert ^{q} \biggr]^{\frac{1}{q}}. \end{aligned}$$

*Especially if we choose* $m=1=k$ *and* $\lambda=0$ *or* $\lambda=1$, *we can get Theorem *7 *in* [38].

### Corollary 3.4

*In Theorem* 3.1, *if we put* $h(t)=1$, *then we obtain the following inequality for* $(m,P)$-*convex functions*:

$$\begin{aligned} & \bigl\vert \mathcal{T}_{k,\mu}(m,\lambda,r) \bigr\vert \\ &\quad \leq\frac{2k}{\mu+k}\biggl[1-\frac{1}{2^{\frac{\mu}{k}}}\biggr] \biggl[ \bigl\vert f'\bigl(\lambda a+m(1-\lambda)r\bigr) \bigr\vert ^{q}+m \biggl\vert f'\biggl(\lambda r+(1-\lambda)\frac{a}{m} \biggr) \biggr\vert ^{q}\biggr]^{\frac{1}{q}}. \end{aligned}$$

*Especially if we choose* $m=1$ *and* $\lambda=1$ *or* $\lambda=0$, *we have*

$$\begin{aligned} & \biggl\vert -\frac{f(a)+f(r)}{2}+\frac{\Gamma_{k}(\mu+k)}{2(r-a)^{\frac{\mu }{k}}} \bigl[{}_{k}J^{\mu}_{a^{+}}f(r)+{}_{k}J^{\mu}_{r^{-}}f(a) \bigr] \biggr\vert \\ &\quad \leq\frac{k(r-a)}{\mu+k}\biggl[1-\frac{1}{2^{\frac{\mu}{k}}}\biggr] \bigl[ \bigl\vert f'(r) \bigr\vert ^{q}+ \bigl\vert f'(a) \bigr\vert ^{q}\bigr]^{\frac{1}{q}}. \end{aligned}$$

### Corollary 3.5

*In Theorem* 3.1, *if we take* $h(t)=t^{-s}$, $s\in(0,1)$, *then we get the following inequality for* $(m,s)$-*Godunova*-*Liven*-*Dragomir convex functions*:

$$\begin{aligned} & \bigl\vert \mathcal{T}_{k,\mu}(m,\lambda,r) \bigr\vert \\ &\quad\leq\biggl[\frac{2k}{\mu+k}\biggl(1-\frac{1}{2^{\frac{\mu}{k}}} \biggr) \biggr]^{1-\frac{1}{q}} \\ & \qquad{}\times\biggl[\beta\biggl(\frac{1}{2}, 1-s, \frac{\mu}{k}+1\biggr)- \beta \biggl(\frac{1}{2}, \frac{\mu}{k}+1, 1-s\biggr)+ \frac{k}{\mu+(1-s)k} \bigl(1-2^{\frac{sk-\mu}{k}}\bigr)\biggr]^{\frac{1}{q}} \\ &\qquad{} \times\biggl[ \bigl\vert f'\bigl(\lambda a+m(1-\lambda)r\bigr) \bigr\vert ^{q}+m \biggl\vert f'\biggl(\lambda r+(1- \lambda)\frac{a}{m}\biggr) \biggr\vert ^{q} \biggr]^{\frac{1}{q}}. \end{aligned}$$

*Especially if we put* $m=1$ *and* $\lambda=1$ *or* $\lambda=0$, *we get*

$$\begin{aligned} & \biggl\vert -\frac{f(a)+f(r)}{r-a}+\frac{\Gamma_{k}(\mu+k)}{(r-a)^{\frac{\mu }{k}+1}} \bigl[{}_{k}J^{\mu}_{a^{+}}f(r)+{}_{k}J^{\mu}_{r^{-}}f(a) \bigr] \biggr\vert \\ &\quad\leq\biggl[\frac{2k}{\mu+k}\biggl(1-\frac{1}{2^{\frac{\mu}{k}}} \biggr) \biggr]^{1-\frac{1}{q}} \\ &\qquad{} \times\biggl[\beta\biggl(\frac{1}{2}, 1-s, \frac{\mu}{k}+1\biggr)- \beta \biggl(\frac{1}{2}, \frac{\mu}{k}+1, 1-s\biggr)+ \frac{k}{\mu+(1-s)k} \bigl(1-2^{\frac{sk-\mu}{k}}\bigr)\biggr]^{\frac{1}{q}} \\ &\qquad{} \times\bigl[ \bigl\vert f'(a) \bigr\vert ^{q}+ \bigl\vert f'(r) \bigr\vert ^{q}\bigr]^{\frac{1}{q}}. \end{aligned}$$

### Corollary 3.6

*In Theorem* 3.1, *if we choose* $h(t)=t(1-t)$, *then we obtain the following inequality for* $(m,tgs)$-*convex functions*:

$$\begin{aligned} \bigl\vert \mathcal{T}_{k,\mu}(m,\lambda,r) \bigr\vert \leq{} &\biggl[\frac{2k}{\mu+k}\biggl(1-\frac{1}{2^{\frac{\mu}{k}}}\biggr) \biggr]^{1-\frac{1}{q}} \biggl[\frac{4k^{2}-2^{-\frac{\mu}{k}}(k\mu+4k^{2})}{2(\mu+2k)(\mu+3k)} \biggr]^{\frac{1}{q}} \\ &{} \times\biggl[ \bigl\vert f'\bigl(\lambda a+m(1-\lambda)r\bigr) \bigr\vert ^{q}+m \biggl\vert f'\biggl(\lambda r+(1- \lambda)\frac{a}{m}\biggr) \biggr\vert ^{q} \biggr]^{\frac{1}{q}}. \end{aligned}$$

*Especially if we put* $m=1$ *and* $\lambda=1$ *or* $\lambda=0$, *we get*

$$\begin{aligned} & \biggl\vert -\frac{f(a)+f(r)}{r-a}+\frac{\Gamma_{k}(\mu+k)}{(r-a)^{\frac{\mu }{k}+1}} \bigl[{}_{k}J^{\mu}_{a^{+}}f(r)+{}_{k}J^{\mu}_{r^{-}}f(a) \bigr] \biggr\vert \\ &\quad\leq\biggl[\frac{2k}{\mu+k}\biggl(1-\frac{1}{2^{\frac{\mu}{k}}}\biggr) \biggr]^{1-\frac{1}{q}} \biggl[\frac{4k^{2}-2^{-\frac{\mu}{k}}(k\mu+4k^{2})}{(\mu+2k) (\mu+3k)} \biggr]^{\frac{1}{q}} \biggl[\frac{ | f'(a)|^{q}+| f'(r) | }{2}^{q}\biggr]^{\frac{1}{q}}. \end{aligned}$$

### Corollary 3.7

*In Theorem* 3.1, *if we choose* $h(t)=\frac{\sqrt{1-t}}{2\sqrt {t}}$, *then we obtain the following inequality for* *m*-*MT*-*convex functions*:

$$\begin{aligned} \bigl\vert \mathcal{T}_{k,\mu}(m,\lambda,r) \bigr\vert \leq{}&\biggl[\frac{2k}{\mu+k}\biggl(1-\frac{1}{2^{\frac{\mu}{k}}}\biggr) \biggr]^{1-\frac{1}{q}} \biggl[\frac{1}{2}\biggl(\beta\biggl( \frac{1}{2}, \frac{1}{2}, \frac{\mu }{k}+\frac{1}{2} \biggr)-\beta\biggl(\frac{1}{2}, \frac{\mu}{k}+\frac{1}{2}, \frac{1}{2}\biggr)\biggr)\biggr]^{\frac{1}{q}} \\ &{} \times\biggl[ \bigl\vert f'\bigl(\lambda a+m(1-\lambda)r\bigr) \bigr\vert ^{q}+m \biggl\vert f'\biggl(\lambda r+(1- \lambda)\frac{a}{m}\biggr) \biggr\vert ^{q} \biggr]^{\frac{1}{q}}. \end{aligned}$$

*Especially if we put* $m=1$ *and* $\lambda=1$ *or* $\lambda=0$, *we get*

$$\begin{aligned} & \biggl\vert -\frac{f(a)+f(r)}{r-a}+\frac{\Gamma_{k}(\mu+k)}{(r-a)^{\frac{\mu }{k}+1}} \bigl[{}_{k}J^{\mu}_{a^{+}}f(r)+{}_{k}J^{\mu}_{r^{-}}f(a) \bigr] \biggr\vert \\ &\quad\leq\biggl[\frac{2k}{\mu+k}\biggl(1-\frac{1}{2^{\frac{\mu}{k}}}\biggr) \biggr]^{1-\frac{1}{q}} \biggl[\beta\biggl(\frac{1}{2}, \frac{1}{2}, \frac{\mu}{k}+\frac{1}{2} \biggr)-\beta\biggl(\frac{1}{2}, \frac{\mu}{k}+\frac{1}{2}, \frac{1}{2} \biggr) \biggr]^{\frac{1}{q}} \\ &\qquad{}\times \biggl[\frac{ \vert f'(a) \vert ^{q}+ \vert f'(r) \vert ^{q}}{2}\biggr]^{\frac{1}{q}}. \end{aligned}$$

Now, we prepare to introduce the second theorem as follows.

### Theorem 3.2

*Under the assumptions of Theorem* 3.1, *the resulting expression exists*:

3.4 $$\begin{aligned} \bigl\vert \mathcal{T}_{k,\mu}(m,\lambda,r) \bigr\vert \leq{}&\biggl[ \int^{\frac{1}{2}}_{0}\bigl((1-t)^{\frac{\mu}{k}q}-t^{\frac {\mu}{k}q} \bigr) \bigl(h(t)+h(1-t)\bigr)\,\mathrm{d}t\biggr]^{\frac{1}{q}} \\ &{} \times\biggl[ \bigl\vert f'\bigl(\lambda a+m(1-\lambda)r\bigr) \bigr\vert ^{q}+m \biggl\vert f'\biggl(\lambda r+(1- \lambda)\frac{a}{m}\biggr) \biggr\vert ^{q} \biggr]^{\frac {1}{q}}, \end{aligned}$$

*where* $\lambda\in[0,1]\setminus\frac{1}{2}$, $k>0$ *and* $\mu>0$.

*Proof* Using Lemma 2.1, Hölder’s inequality and the $(h,m)$-convexity of $\vert f' \vert ^{q}$, we have

$$\begin{aligned} & \bigl\vert \mathcal{T}_{k,\mu}(m,\lambda,r) \bigr\vert \\ &\quad\leq \int^{1}_{0} \bigl\vert (1-t)^{\frac{\mu}{k}}-t^{\frac{\mu}{k}} \bigr\vert \biggl\vert f'\biggl(t \bigl(\lambda a+m(1-\lambda)r\bigr)+m(1-t) \biggl(\lambda r+(1-\lambda) \frac{a}{m}\biggr)\biggr) \biggr\vert \,\mathrm{d}t \\ &\quad\leq\biggl( \int^{1}_{0}1^{p}\,\mathrm{d}t \biggr)^{\frac{1}{p}} \biggl[ \int^{1}_{0} \bigl\vert (1-t)^{\frac{\mu}{k}}-t^{\frac{\mu}{k}} \bigr\vert ^{q} \\ &\qquad{} \times \biggl\vert f'\biggl(t\bigl(\lambda a+m(1-\lambda)r \bigr)+m(1-t) \biggl(\lambda r+(1-\lambda)\frac{a}{m}\biggr)\biggr) \biggr\vert ^{q}\,\mathrm{d}t \biggr]^{\frac{1}{q}} \\ &\quad=\biggl[ \int^{\frac{1}{2}}_{0}\bigl((1-t)^{\frac{\mu}{k}}-t^{\frac{\mu}{k}}\bigr)^{q} \\ &\qquad{} \times \biggl\vert f'\biggl(t\bigl(\lambda a+m(1-\lambda)r \bigr)+m(1-t) \biggl(\lambda r+(1-\lambda)\frac{a}{m}\biggr)\biggr) \biggr\vert ^{q}\,\mathrm{d}t \\ & \qquad{}+ \int^{1}_{\frac{1}{2}}\bigl(t^{\frac{\mu}{k}}-(1-t)^{\frac{\mu}{k}} \bigr)^{q} \\ & \qquad{}\times \biggl\vert f'\biggl(t\bigl(\lambda a+m(1-\lambda)r \bigr)+m(1-t) \biggl(\lambda r+(1-\lambda)\frac{a}{m}\biggr)\biggr) \biggr\vert ^{q}\,\mathrm{d}t \biggr]^{\frac{1}{q}} \\ &\quad\leq\biggl\{ \int^{\frac{1}{2}}_{0}\bigl((1-t)^{\frac{\mu}{k}q}-t^{\frac {\mu}{k}q} \bigr) \\ &\qquad{} \times\biggl[h(t) \bigl\vert f'\bigl(\lambda a+m(1-\lambda)r \bigr)\bigr\vert ^{q}+mh(1-t) \biggl\vert f'\biggl(\lambda r+(1- \lambda)\frac{a}{m}\biggr) \biggr\vert ^{q}\,\mathrm{d}t\biggr] \\ &\qquad{} + \int^{1}_{\frac{1}{2}}\bigl(t^{\frac{\mu}{k}q}-(1-t)^{\frac{\mu }{k}q} \bigr) \\ &\qquad{} \times\biggl[h(t) \bigl\vert f'\bigl(\lambda a+m(1-\lambda)r\bigr) \bigr\vert ^{q}+mh(1-t) \biggl\vert f'\biggl(\lambda r+(1- \lambda)\frac{a}{m}\biggr) \biggr\vert ^{q}\biggr]\,\mathrm {d}t \biggr\} ^{\frac{1}{q}} \\ &\quad=\biggl[ \int^{\frac{1}{2}}_{0}\bigl((1-t)^{\frac{\mu}{k}q}-t^{\frac{\mu }{k}q} \bigr) \bigl(h(t)+h(1-t)\bigr)\,\mathrm{d}t\biggr]^{\frac{1}{q}} \\ &\qquad{} \times\biggl[ \bigl\vert f'\bigl(\lambda a+m(1-\lambda)r\bigr) \bigr\vert ^{q}+m \biggl\vert f' \biggl(\lambda r+(1- \lambda)\frac{a}{m}\biggr) \biggr\vert ^{q} \biggr]^{\frac{1}{q}}. \end{aligned}$$

Here, we use $(A-B)^{q}\leq A^{q}-B^{q}$ for any $A\geq B\geq0$ and $q\geq 1$.

Let us point out some special cases of Theorem 3.2.

### Corollary 3.8

*In Theorem* 3.2, *if we put* $h(t)=t^{s}$, $s\in(0,1]$, *then we get the following inequality for* $(s,m)$-*Breckner convex functions*:

$$\begin{aligned} & \bigl\vert \mathcal{T}_{k,\mu}(m,\lambda,r) \bigr\vert \\ &\quad\leq\biggl[\beta\biggl(\frac{1}{2},s+1,{\frac{\mu}{k}q}+1\biggr)- \beta \biggl(\frac{1}{2},{\frac{\mu}{k}q}+1,s+1\biggr)+ \frac{k}{\mu q+(s+1)k}\bigl(1-2^{-\frac{\mu q+sk}{k}}\bigr)\biggr]^{\frac {1}{q}} \\ & \qquad{}\times\biggl[ \bigl\vert f'\bigl(\lambda a+(1-\lambda)r\bigr) \bigr\vert ^{q}+ m \biggl\vert f'\biggl(\lambda r+(1- \lambda)\frac{a}{m}\biggr) \biggr\vert ^{q} \biggr]^{\frac {1}{q}}. \end{aligned}$$

*Especially if we put* $m=1$ *and* $\lambda=0$ *or* $\lambda=1$, *we have*

$$\begin{aligned} & \biggl\vert -\frac{f(a)+f(r)}{r-a}+\frac{\Gamma_{k}(\mu +k)}{(r-a)^{\frac{\mu}{k}+1}} \bigl[{}_{k}J^{\mu }_{a^{+}}f(r)+{}_{k}J^{\mu}_{r^{-}}f(a) \bigr] \biggr\vert \\ &\quad\leq\biggl[\beta\biggl(\frac{1}{2},s+1,{\frac{\mu}{k}q}+1\biggr)- \beta \biggl(\frac{1}{2},{\frac{\mu}{k}q}+1,s+1\biggr)+ \frac{k}{\mu q+(s+1)k}\bigl(1-2^{-\frac{\mu q+sk}{k}}\bigr)\biggr]^{\frac {1}{q}} \\ &\qquad{} \times\bigl[ \bigl\vert f'(r) \bigr\vert ^{q}+ \bigl\vert f'(a) \bigr\vert ^{q}\bigr]^{\frac{1}{q}}. \end{aligned}$$

### Corollary 3.9

*In Theorem* 3.2, *if we take* $h(t)=t^{-s}$, $s\in(0,1]$, *then we get the following inequality for* $(m,s)$-*Godunova*-*Levin*-*Dragomir convex functions*:

$$\begin{aligned} & \bigl\vert \mathcal{T}_{k,\mu}(m,\lambda,r) \bigr\vert \\ &\quad\leq\biggl[\beta\biggl(\frac{1}{2}, 1-s, \frac{\mu}{k}+1\biggr)- \beta \biggl(\frac{1}{2}, \frac{\mu}{k}+1, 1-s\biggr) + \frac{k}{k(1-s)+\mu}\bigl(1-2^{\frac{sk-\mu}{k}}\bigr)\biggr]^{\frac {1}{q}} \\ &\qquad{} \times\biggl[ \bigl\vert f'\bigl(\lambda a+m(1-\lambda)r\bigr) \bigr\vert ^{q}+m \biggl\vert f'\biggl(\lambda r+(1- \lambda)\frac{a}{m}\biggr) \biggr\vert ^{q} \biggr]^{\frac {1}{q}}. \end{aligned}$$

*Especially if we take* $m=1$ *and* $\lambda=0$ *or* $\lambda=1$, *we have*

$$\begin{aligned} & \biggl\vert -\frac{f(a)+f(r)}{r-a}+\frac{\Gamma_{k}(\mu +k)}{(r-a)^{\frac{\mu}{k}+1}} \bigl[{}_{k}J^{\mu }_{a^{+}}f(r)+{}_{k}J^{\mu}_{r^{-}}f(a) \bigr] \biggr\vert \\ &\quad\leq\biggl[\beta\biggl(\frac{1}{2}, 1-s, \frac{\mu}{k}+1\biggr)- \beta \biggl(\frac{1}{2}, \frac{\mu}{k}+1, 1-s\biggr) + \frac{k}{k(1-s)+\mu}\bigl(1-2^{\frac{sk-\mu}{k}}\bigr)\biggr]^{\frac {1}{q}} \\ &\qquad{} \times\bigl[ \bigl\vert f'(r) \bigr\vert ^{q}+ \bigl\vert f'(a) \bigr\vert ^{q}\bigr]^{\frac{1}{q}}. \end{aligned}$$

### Corollary 3.10

*In Theorem* 3.2, *if we put* $h(t)=t(1-t)$, *then we get the following inequality for* $(m,tgs)$-*convex functions*:

$$\begin{aligned} &\bigl\vert \mathcal{T}_{k,\mu}(m,\lambda,r) \bigr\vert \\ &\quad \leq\biggl[\frac{2k^{2}-(\frac{1}{2})^{\frac{\mu q+k}{k}}(4k^{2}+kuq)}{(\mu q+2k)(\mu q+3k)}\biggr]^{\frac{1}{q}} \\ &\qquad {}\times\biggl[ \bigl\vert f'\bigl(\lambda a+m(1-\lambda)r\bigr) \bigr\vert ^{q}+m \biggl\vert f'\biggl(\lambda r+(1- \lambda)\frac{a}{m}\biggr) \biggr\vert ^{q} \biggr]^{\frac{1}{q}}. \end{aligned}$$

*Especially if we put* $m=1$ *and* $\lambda=0$ *or* $\lambda=1$, *we have*

$$\begin{aligned} & \biggl\vert -\frac{f(a)+f(r)}{r-a}+\frac{\Gamma_{k}(\mu +k)}{(r-a)^{\frac{\mu}{k}+1}} \bigl[{}_{k}J^{\mu }_{a^{+}}f(r)+{}_{k}J^{\mu}_{r^{-}}f(a) \bigr] \biggr\vert \\ &\quad\leq\biggl[\frac{2k^{2}-(\frac{1}{2})^{\frac{\mu q+k}{k}}(4k^{2}+kuq)}{(\mu q+2k)(\mu q+3k)}\biggr]^{\frac{1}{q}} \bigl[ \bigl\vert f'(r) \bigr\vert ^{q}+ \bigl\vert f'(a) \bigr\vert ^{q}\bigr]^{\frac{1}{q}}. \end{aligned}$$

### Corollary 3.11

*In Theorem* 3.2, *if we put* $h(t)=\frac{\sqrt{1-t}}{2\sqrt{t}}$, *then we get the following inequality for* *m*-*MT*-*convex functions*:

$$\begin{aligned} & \bigl\vert \mathcal{T}_{k,\mu}(m,\lambda,r) \bigr\vert \\ &\quad\leq\biggl[\beta\biggl(\frac{1}{2},\frac{1}{2},\frac{\mu}{k}q+ \frac {1}{2}\biggr)-\beta\biggl(\frac{1}{2},\frac{\mu}{k}q+ \frac{1}{2},\frac {1}{2}\biggr)\biggr]^{\frac{1}{q}} \\ &\qquad {}\times\biggl[\frac{ \vert f'(\lambda a+m(1-\lambda)r) \vert ^{q}+m \vert f'(\lambda r+(1-\lambda)\frac{a}{m}) \vert ^{q}}{2}\biggr]^{\frac{1}{q}}. \end{aligned}$$

*Especially if we put* $m=1$ *and* $\lambda=0$ *or* $\lambda=1$, *we have*

$$\begin{aligned} & \biggl\vert -\frac{f(a)+f(r)}{r-a}+\frac{\Gamma_{k}(\mu +k)}{(r-a)^{\frac{\mu}{k}+1}} \bigl[{}_{k}J^{\mu }_{a^{+}}f(r)+{}_{k}J^{\mu}_{r^{-}}f(a) \bigr] \biggr\vert \\ &\quad\leq\biggl[\beta\biggl(\frac{1}{2},\frac{1}{2}, \frac{\mu}{k}q+\frac {1}{2}\biggr)-\beta\biggl(\frac{1}{2}, \frac{\mu}{k}q+\frac{1}{2},\frac {1}{2}\biggr) \biggr]^{\frac{1}{q}} \biggl[\frac{ \vert f'(r) \vert ^{q}+ \vert f'(a) \vert ^{q}}{2}\biggr]^{\frac{1}{q}}. \end{aligned}$$

Now, we are ready to state the third theorem in this section.

### Theorem 3.3

*Let* $h:J\subseteq\mathbb{R}\rightarrow\mathbb{R}\ ([0,1]\subseteq J)$ *be a non*-*negative function*, *and let* $f:I\subseteq\mathbb{R}\rightarrow\mathbb{R}$ *be a differentiable mapping on* $I^{o}$ *along with* $a,r\in I$, $0\leq a < mr$, *for some fixed* $m\in(0,1]$. *If* $f'\in L[a,mr]$ *and* $\vert f' \vert ^{q}$ *for* $q>1$ *is* $(h,m)$-*convex on* $[a,mr]$, *then the following inequality holds*:

3.5 $$\begin{aligned} & \bigl\vert \mathcal{T}_{k,\mu}(m,\lambda,r) \bigr\vert \\ &\quad\leq\biggl[\frac{2k}{\mu p+k}\biggl(1-\frac{1}{2^{\frac{\mu}{k}p}} \biggr) \biggr]^{\frac{1}{p}} \\ & \qquad{}\times\biggl\{ \int^{1}_{0}\biggl[h(t) \bigl\vert f'\bigl(\lambda a+m(1-\lambda )r\bigr) \bigr\vert ^{q} \\ &\qquad{}+mh(1-t) \biggl\vert f'\biggl(\lambda r+(1-\lambda) \frac{a}{m} \biggr) \biggr\vert ^{q}\biggr]\,\mathrm{d}t\biggr\} ^{\frac{1}{q}}, \end{aligned}$$

*where* $\frac{1}{p}+\frac{1}{q}=1$, $\mu>0$, $k>0$ *and* $\lambda\in [0,1]\setminus\frac{1}{2}$.

### Proof

Applying Lemma 2.1, Hölder’s inequality and the $(h,m)$-convexity of $\vert f' \vert ^{q}$, we have

$$\begin{aligned} & \bigl\vert \mathcal{T}_{k,\mu}(m,\lambda,r) \bigr\vert \\ &\quad\leq \int^{1}_{0} \bigl\vert (1-t)^{\frac{\mu}{k}}-t^{\frac{\mu}{k}} \bigr\vert \biggl\vert f'\biggl(t \bigl(\lambda a+m(1-\lambda)r\bigr)+m(1-t) \biggl(\lambda r+(1-\lambda) \frac{a}{m}\biggr)\biggr) \biggr\vert \,\mathrm{d}t \\ &\quad\leq\biggl[ \int^{1}_{0} \bigl\vert (1-t)^{\frac{\mu}{k}}-t^{\frac{\mu}{k}} \bigr\vert ^{p}\biggr]^{\frac{1}{p}} \\ & \qquad{}\times\biggl[ \biggl\vert f'\biggl(t\bigl(\lambda a+m(1- \lambda)r\bigr)+m(1-t) \biggl(\lambda r+(1-\lambda)\frac{a}{m}\biggr) \biggr) \biggr\vert ^{q}\,\mathrm{d}t \biggr]^{\frac{1}{q}} \\ &\quad=\biggl[ \int^{\frac{1}{2}}_{0}\bigl((1-t)^{\frac{\mu}{k}}-t^{\frac{\mu}{k}}\bigr)^{p}\,\mathrm{d}t + \int^{1}_{\frac{1}{2}}\bigl(t^{\frac{\mu}{k}} -(1-t)^{\frac{\mu}{k}}\bigr)^{p}\,\mathrm{d}t \biggr]^{\frac{1}{p}} \\ & \qquad{}\times\biggl\{ \int^{1}_{0}\biggl[h(t) \bigl\vert f'\bigl(\lambda a+m(1-\lambda )r\bigr) \bigr\vert ^{q}\\ &\qquad{}+mh(1-t) \biggl\vert f'\biggl(\lambda r+(1-\lambda) \frac{a}{m} \biggr) \biggr\vert ^{q}\biggr]\,\mathrm{d}t\biggr\} ^{\frac{1}{q}} \\ &\quad\leq\biggl[ \int^{\frac{1}{2}}_{0}\bigl((1-t)^{\frac{\mu}{k}p}-t^{\frac {\mu}{k}p} \bigr)\,\mathrm{d}t+ \int^{1}_{\frac{1}{2}}\bigl(t^{\frac{\mu}{k}p} -(1-t)^{\frac{\mu}{k}p}\bigr)\,\mathrm{d}t\biggr]^{\frac{1}{p}} \\ &\qquad{} \times\biggl\{ \int^{1}_{0}\biggl[h(t) \bigl\vert f'\bigl(\lambda a+m(1-\lambda )r\bigr) \bigr\vert ^{q}+mh(1-t) \biggl\vert f'\biggl(\lambda r+(1-\lambda) \frac{a}{m} \biggr) \biggr\vert ^{q}\biggr]\,\mathrm{d}t\biggr\} ^{\frac{1}{q}} \\ &\quad=\biggl[\frac{2k}{\mu p+k}\biggl(1-\frac{1}{2^{\frac{\mu}{k}p}} \biggr) \biggr]^{\frac{1}{p}} \\ &\qquad{} \times\biggl\{ \int^{1}_{0}\biggl[h(t) \bigl\vert f'\bigl(\lambda a+m(1-\lambda )r\bigr) \bigr\vert ^{q}+mh(1-t) \biggl\vert f'\biggl(\lambda r+(1-\lambda) \frac{a}{m} \biggr) \biggr\vert ^{q}\biggr]\,\mathrm{d}t\biggr\} ^{\frac{1}{q}}. \end{aligned}$$

Here, we use the fact that $(A-B)^{q}\leq A^{q}-B^{q}$ for any $A\geq B\geq 0$ and $q\geq1$, which completes the proof. □

Now, we point out some special cases of Theorem 3.3.

### Corollary 3.12

*In Theorem* 3.3, *if we choose* $h(t)=t$ *and* $r=b$, *then we obtain the following inequality for* *m*-*convex functions*:

$$\begin{aligned} & \bigl\vert \mathcal{T}_{k,\mu}(m,\lambda,b) \bigr\vert \\ &\quad\leq\biggl[\frac{2k}{\mu p+k}\biggl(1-\frac{1}{2^{\frac{\mu}{k}p}} \biggr) \biggr]^{\frac{1}{p}}\biggl[\frac{ \vert f'(\lambda a+(1-\lambda)b ) \vert ^{q}+ m \vert f'(\lambda b+(1-\lambda)\frac{a}{m}) \vert ^{q}}{2} \biggr]^{\frac{1}{q}}. \end{aligned}$$

*Especially if we put* $k=1$, *we obtain Theorem *3.3 *in* [35]. *Further*, *if we put* $m=1$, *we obtain Theorem *2.6 *in* [12].

### Corollary 3.13

*In Theorem* 3.3, *if we choose* $h(t)=t$, $m=1$ *and* $\lambda=0$ *or* $\lambda=1$, *then we obtain the following inequality for convex functions*:

$$\begin{aligned} & \biggl\vert -\frac{f(a)+f(r)}{r-a}+\frac{\Gamma_{k}(\mu+k)}{(r-a)^{\frac{\mu }{k}+1}} \bigl[{}_{k}J^{\mu}_{a^{+}}f(r)+{}_{k}J^{\mu}_{r^{-}}f(a) \bigr] \biggr\vert \\ &\quad\leq\biggl[\frac{2k}{\mu p+k}\biggl(1-\frac{1}{2^{\frac{\mu}{k}p}} \biggr) \biggr]^{\frac{1}{p}}\biggl[\frac{ \vert f'(r) \vert ^{q}+ \vert f'(a) \vert ^{q}}{2}\biggr]^{\frac{1}{q}}. \end{aligned}$$

### Remark 3.2

In Corollary 3.13,

- if we take $k=1$ and $r=b$, we can get Corollary 2.7 in [12],
- if we take $k=1=\mu$ and $r=b$, we can get Corollary 2.8 in [12].

### Corollary 3.14

*In Theorem* 3.3, *if we choose* $h(t)=t^{s}$, $s\in(0,1]$, *then we obtain the following inequality for* $(s,m)$-*Breckner convex functions*:

$$\begin{aligned} & \bigl\vert \mathcal{T}_{k,\mu}(m,\lambda,r) \bigr\vert \\ &\quad\leq\biggl[\frac{2k}{\mu p+k}\biggl(1-\frac{1}{2^{\frac{\mu}{k}p}} \biggr) \biggr]^{\frac{1}{p}}\biggl[\frac{ \vert f'(\lambda a+(1-\lambda)r ) \vert ^{q}+ m \vert f'(\lambda r+(1-\lambda)\frac{a}{m}) \vert ^{q}}{s+1} \biggr]^{\frac{1}{q}}. \end{aligned}$$

*Especially if we put* $m=1$ *and* $\lambda=0$ *or* $\lambda=1$, *then we have*

$$\begin{aligned} & \biggl\vert -\frac{f(a)+f(r)}{r-a}+\frac{\Gamma_{k}(\mu+k)}{(r-a)^{\frac{\mu }{k}+1}} \bigl[{}_{k}J^{\mu}_{a^{+}}f(r)+{}_{k}J^{\mu}_{r^{-}}f(a) \bigr] \biggr\vert \\ &\quad\leq\biggl[\frac{2k}{\mu p+k}\biggl(1-\frac{1}{2^{\frac{\mu}{k}p}} \biggr) \biggr]^{\frac{1}{p}}\biggl[\frac{ \vert f'(a) \vert ^{q}+ \vert f'(r) \vert ^{q}}{s+1}\biggr]^{\frac{1}{q}}. \end{aligned}$$

### Corollary 3.15

*In Theorem* 3.3, *if we put* $h(t)=1$, *then we obtain the following inequality for* $(m,P)$-*convex functions*:

$$\begin{aligned} & \bigl\vert \mathcal{T}_{k,\mu}(m,\lambda,r) \bigr\vert \\ &\quad\leq\biggl[\frac{2k}{\mu p+k}\biggl(1-\frac{1}{2^{\frac{\mu}{k}p}} \biggr) \biggr]^{\frac{1}{p}}\biggl[ \bigl\vert f'\bigl(\lambda a+(1-\lambda)r\bigr) \bigr\vert ^{q}+ m \biggl\vert f'\biggl(\lambda r+(1-\lambda)\frac{a}{m}\biggr) \biggr\vert ^{q}\biggr]^{\frac{1}{q}}. \end{aligned}$$

*Especially if we put* $m=1$ *and* $\lambda=1$ *or* $\lambda=0$, *we have*

$$\begin{aligned} & \biggl\vert -\frac{f(a)+f(r)}{r-a}+\frac{\Gamma_{k}(\mu+k)}{(r-a)^{\frac{\mu }{k}+1}} \bigl[{}_{k}J^{\mu}_{a^{+}}f(r)+{}_{k}J^{\mu}_{r^{-}}f(a) \bigr] \biggr\vert \\ &\quad\leq\biggl[\frac{2k}{\mu p+k}\biggl(1-\frac{1}{2^{\frac{\mu}{k}p}} \biggr) \biggr]^{\frac{1}{p}}\bigl[ \bigl\vert f'(r) \bigr\vert ^{q}+ \bigl\vert f'(a) \bigr\vert ^{q} \bigr]^{\frac{1}{q}}. \end{aligned}$$

### Corollary 3.16

*In Theorem* 3.3, *if we take* $h(t)=t^{-s}$, $s\in(0,1)$, *then we obtain the following inequality for* $(s,m)$-*Godunova*-*Levin*-*Dragomir convex functions*:

$$\begin{aligned} & \bigl\vert \mathcal{T}_{k,\mu}(m,\lambda,r) \bigr\vert \\ &\quad\leq\biggl[\frac{2k}{\mu p+k}\biggl(1-\frac{1}{2^{\frac{\mu}{k}p}} \biggr) \biggr]^{\frac{1}{p}}\biggl[\frac{ \vert f'(\lambda a+(1-\lambda)r ) \vert ^{q}+ m \vert f'(\lambda r+(1-\lambda)\frac{a}{m}) \vert ^{q}}{1-s} \biggr]^{\frac{1}{q}}. \end{aligned}$$

*Especially if we take* $m=1$ *and* $\lambda=1$ *or* $\lambda=0$, *we have*

$$\begin{aligned} & \biggl\vert -\frac{f(a)+f(r)}{r-a}+\frac{\Gamma_{k}(\mu+k)}{(r-a)^{\frac{\mu }{k}+1}} \bigl[{}_{k}J^{\mu}_{a^{+}}f(r)+{}_{k}J^{\mu}_{r^{-}}f(a) \bigr] \biggr\vert \\ &\quad\leq\biggl[\frac{2k}{\mu p+k}\biggl(1-\frac{1}{2^{\frac{\mu}{k}p}} \biggr) \biggr]^{\frac{1}{p}} \biggl[\frac{ \vert f'(r) \vert ^{q}+ \vert f'(a) \vert ^{q}}{1-s}\biggr]^{\frac{1}{q}}. \end{aligned}$$

### Corollary 3.17

*In Theorem* 3.3, *if we put* $h(t)=\frac{\sqrt{1-t}}{2\sqrt{t}}$, *then we obtain the following inequality for* *m*-*MT*-*convex functions*:

$$\begin{aligned} & \bigl\vert \mathcal{T}_{k,\mu}(m,\lambda,r) \bigr\vert \\ &\quad\leq\biggl(\frac{\pi}{4}\biggr)^{\frac{1}{q}}\biggl[ \frac{2k}{\mu p+k} \biggl(1-\frac{1}{2^{\frac{\mu}{k}p}}\biggr)\biggr]^{\frac{1}{p}} \\ &\qquad{}\times\biggl[ \bigl\vert f' \bigl(\lambda a+(1-\lambda)r\bigr) \bigr\vert ^{q}+ m \biggl\vert f'\biggl(\lambda r+(1- \lambda )\frac{a}{m}\biggr) \biggr\vert ^{q} \biggr]^{\frac{1}{q}}. \end{aligned}$$

*Especially if we put* $m=1$ *and* $\lambda=0$ *or* $\lambda=1$, *we have*

$$\begin{aligned} & \biggl\vert -\frac{f(a)+f(r)}{r-a}+\frac{\Gamma_{k}(\mu +k)}{(r-a)^{\frac{\mu}{k}+1}} \bigl[{}_{k}J^{\mu }_{a^{+}}f(r)+{}_{k}J^{\mu}_{r^{-}}f(a) \bigr] \biggr\vert \\ &\quad \leq\biggl(\frac{\pi}{4}\biggr)^{\frac{1}{q}}\biggl[\frac{2k}{\mu p+k} \biggl(1-\frac{1}{2^{\frac{\mu}{k}p}}\biggr)\biggr]^{\frac{1}{p}}\bigl[ \bigl\vert f'(r) \bigr\vert + \bigl\vert f'(a) \bigr\vert \bigr]^{\frac{1}{q}}. \end{aligned}$$

### Corollary 3.18

*In Theorem* 3.3, *if we choose* $h(t)=t(1-t)$, *then we obtain the following inequality for* $(m,tgs)$-*convex functions*:

$$\begin{aligned} & \bigl\vert \mathcal{T}_{k,\mu}(m,\lambda,r) \bigr\vert \\ &\quad \leq\biggl(\frac{1}{6}\biggr)^{\frac{1}{q}}\biggl[ \frac{2k}{\mu p+k} \biggl(1-\frac{1}{2^{\frac{\mu}{k}p}}\biggr)\biggr]^{\frac{1}{p}} \biggl[ \bigl\vert f' \bigl(\lambda a+(1-\lambda)r\bigr) \bigr\vert ^{q}+ m \biggl\vert f'\biggl(\lambda r+(1- \lambda )\frac{a}{m}\biggr) \biggr\vert ^{q} \biggr]^{\frac{1}{q}}. \end{aligned}$$

*Especially if we choose* $m=1$ *and* $\lambda=0$ *or* $\lambda=1$, *we have*

$$\begin{aligned} & \biggl\vert -\frac{f(a)+f(r)}{r-a}+\frac{\Gamma_{k}(\mu +k)}{(r-a)^{\frac{\mu}{k}+1}} \bigl[{}_{k}J^{\mu }_{a^{+}}f(r)+{}_{k}J^{\mu}_{r^{-}}f(a) \bigr] \biggr\vert \\ &\quad \leq\biggl(\frac{1}{6}\biggr)^{\frac{1}{q}}\biggl[ \frac{2k}{\mu p+k} \biggl(1-\frac{1}{2^{\frac{\mu}{k}p}}\biggr)\biggr]^{\frac{1}{p}} \bigl[ \bigl\vert f'(r) \bigr\vert ^{q}+ \bigl\vert f'(a) \bigr\vert ^{q}\bigr]^{\frac{1}{q}}. \end{aligned}$$

## *k*-fractional inequalities for $(\alpha,m)$-convex functions

Using Lemma 2.1 again, we state the following theorems.

### Theorem 4.1

*Let* $f:I\subseteq\mathbb{R}\rightarrow\mathbb{R}$ *be a differentiable mapping on* $I^{o}$ *along with* $a,r\in I$ *and* $0\leq a < mr$. *If* $\vert f' \vert ^{q}$ *for* $q\geq1$ *is* $(\alpha,m)$-*convex on* $[a,mr]$ *and* $f'\in L^{1}[a,mr]$. *Then the following inequality for* *k*-*fractional integrals holds*:

4.1 $$\begin{aligned} & \bigl\vert \mathcal{T}_{k,\mu}(m,\lambda,r) \bigr\vert \\ &\quad\leq\biggl[\frac{2k}{\mu+k}\biggl(1-\frac{1}{2^{\frac{\mu}{k}}}\biggr) \biggr]^{1-\frac{1}{q}} \biggl\{ \biggl[\beta\biggl(\frac{1}{2},\alpha+1, \frac{\mu}{k}+1\biggr)-\beta \biggl(\frac{1}{2},\frac{\mu}{k}+1, \alpha+1\biggr) \\ &\qquad{} +\frac{k}{\mu+(\alpha+1)k}-\frac{k}{\mu+(\alpha+1)k}\biggl(\frac {1}{2} \biggr)^{\frac{\mu+\alpha k}{k}}\biggr] \bigl\vert f' \bigl(\lambda a+m(1-\lambda)r\bigr) \bigr\vert ^{q} \\ &\qquad{} -\biggl[\beta\biggl(\frac{1}{2},\alpha+1,\frac{\mu}{k}+1\biggr)- \beta \biggl(\frac{1}{2},\frac{\mu}{k}+1,\alpha+1\biggr)+ \frac{k}{\mu+(\alpha+1)k} \\ &\qquad{} -\frac{k}{\mu+(\alpha+1)k}\biggl(\frac{1}{2}\biggr)^{\frac{\mu+\alpha k}{k}} \\ &\qquad{}+\frac{2k}{\mu+k}\biggl(\frac{1}{2}\biggr) ^{\frac{\mu}{k}}-\frac{2k}{\mu +k}\biggr] m \biggl\vert f'\biggl(\lambda r+(1-\lambda)\frac{a}{m}\biggr) \biggr\vert ^{q}\biggr\} ^{\frac {1}{q}}, \end{aligned}$$

*where* $\lambda\in(0,1]\backslash\frac{1}{2}$, $k>0$ *and* $\mu>0$.

### Proof

Using Lemma 2.1, the power mean inequality and the $(\alpha,m)$-convexity of $\vert f' \vert ^{q}$, we have

$$\begin{aligned} & \bigl\vert \mathcal{T}_{k,\mu}(m,\lambda,r) \bigr\vert \\ &\quad\leq \int^{1}_{0} \bigl\vert (1-t)^{\frac{\mu}{k}}-t^{\frac{\mu}{k}} \bigr\vert \biggl\vert f'\biggl(t \bigl(\lambda a+m(1-\lambda)r\bigr)+m(1-t) \biggl(\lambda r+(1-\lambda) \frac{a}{m}\biggr)\biggr) \biggr\vert \,\mathrm{d}t \\ &\quad\leq\biggl[ \int^{1}_{0} \bigl\vert (1-t)^{\frac{\mu}{k}}-t^{\frac{\mu}{k}} \bigr\vert \,\mathrm{d}t\biggr]^{1-\frac{1}{q}} \\ &\qquad{} \times\biggl[ \int^{1}_{0} \bigl\vert (1-t)^{\frac{\mu}{k}}-t^{\frac{\mu}{k}} \bigr\vert \biggl\vert f'\biggl(t \bigl(\lambda a+m(1-\lambda)r\bigr)+m(1-t) \biggl(\lambda r+(1-\lambda ) \frac{a}{m}\biggr)\biggr) \biggr\vert ^{q}\,\mathrm{d}t \biggr]^{\frac{1}{q}} \\ &\quad\leq\biggl[ \int^{\frac{1}{2}}_{0}\bigl((1-t)^{\frac{\mu}{k}}-t^{\frac{\mu}{k}}\bigr)\,\mathrm{d}t+ \int^{1}_{\frac{1}{2}} \bigl(t^{\frac{\mu}{k}}-(1-t)^{\frac{\mu}{k}}\bigr)\,\mathrm{d}t\biggr]^{1-\frac {1}{q}} \\ & \qquad{}\times\biggl\{ \int^{1}_{0} \bigl\vert (1-t)^{\frac{\mu}{k}}-t^{\frac{\mu}{k}} \bigr\vert \\ &\qquad{} \times\biggl[t^{\alpha}\bigl\vert f'\bigl(\lambda a+m(1-\lambda)r\bigr) \bigr\vert ^{q}+m\bigl(1-t^{\alpha}\bigr) \biggl\vert f'\biggl(\lambda r+(1-\lambda)\frac{a}{m} \biggr) \biggr\vert ^{q}\biggr]\,\mathrm{d}t\biggr\} ^{\frac{1}{q}} \\ &\quad=\biggl[\frac{2k}{\mu+k}\biggl(1-\frac{1}{2^{\frac{\mu}{k}}}\biggr) \biggr]^{1-\frac{1}{q}} \biggl\{ \biggl[\beta\biggl(\frac{1}{2},\alpha+1, \frac{\mu}{k}+1\biggr)-\beta \biggl(\frac{1}{2},\frac{\mu}{k}+1, \alpha+1\biggr) \\ &\qquad{} +\frac{k}{\mu+(\alpha+1)k}-\frac{k}{\mu+(\alpha+1)k}\biggl(\frac {1}{2} \biggr)^{\frac{\mu+\alpha k}{k}}\biggr] \bigl\vert f' \bigl(\lambda a+m(1-\lambda)r\bigr) \bigr\vert ^{q} \\ &\qquad{} -\biggl[\beta\biggl(\frac{1}{2},\alpha+1,\frac{\mu}{k}+1\biggr)- \beta \biggl(\frac{1}{2},\frac{\mu}{k}+1,\alpha+1\biggr)+ \frac{k}{\mu+(\alpha+1)k} \\ & \qquad{}-\frac{k}{\mu+(\alpha+1)k}\biggl(\frac{1}{2}\biggr)^{\frac{\mu+\alpha k}{k}} +\frac{2k}{\mu+k}\biggl(\frac{1}{2}\biggr)^{\frac{\mu}{k}}-\frac{2k}{\mu +k}\biggr] m \biggl\vert f'\biggl(\lambda r+(1-\lambda)\frac{a}{m}\biggr) \biggr\vert ^{q}\biggr\} ^{\frac{1}{q}}, \end{aligned}$$

which completes the proof. □

### Theorem 4.2

*Under the assumptions of Theorem* 4.1, *the following inequality for* *k*-*fractional integrals holds*:

4.2 $$\begin{aligned} & \bigl\vert \mathcal{T}_{k,\mu}(m,\lambda,r) \bigr\vert \\ &\quad\leq\biggl\{ \biggl[\beta\biggl(\frac{1}{2},\alpha+1, \frac{\mu}{k}q+1 \biggr)-\beta\biggl(\frac{1}{2},\frac{\mu}{k}q+1, \alpha+1\biggr) \\ &\qquad{} +\frac{k}{\mu q+(\alpha+1)k}-\frac{k}{\mu q+(\alpha+1)k}\biggl(\frac {1}{2} \biggr)^{\frac{\mu q+\alpha k}{k}}\biggr] \bigl\vert f' \bigl(\lambda a+m(1-\lambda)r\bigr) \bigr\vert ^{q} \\ & \qquad{}-\biggl[\beta\biggl(\frac{1}{2},\alpha+1,\frac{\mu}{k}q+1\biggr)- \beta \biggl(\frac{1}{2},\frac{\mu}{k}q+1,\alpha+1\biggr)- \frac{k}{\mu q+(\alpha +1)k}\biggl(\frac{1}{2}\biggr)^{\frac{\mu q+\alpha k}{k}} \\ & \qquad{}+\frac{k}{\mu q+(\alpha+1)k}-\frac{2k}{\mu q+k}+\frac{2k}{\mu q+k}\biggl( \frac{1}{2}\biggr)^{\frac{\mu q}{k}}\biggr] m \biggl\vert f'\biggl(\lambda r+m(1-\lambda)\frac{a}{m}\biggr) \biggr\vert ^{q} \biggr\} ^{\frac{1}{q}}, \end{aligned}$$

*where* $\lambda\in(0,1]\backslash\frac{1}{2}$, $k>0$ *and* $\mu>0$.

### Proof

By making use of Lemma 2.1, Hölder’s inequality and the $(\alpha,m)$-convexity of $\vert f' \vert ^{q}$, we get

$$\begin{aligned} & \bigl\vert \mathcal{T}_{k,\mu}(m,\lambda,r) \bigr\vert \\ &\quad\leq \int^{1}_{0} \bigl\vert (1-t)^{\frac{\mu}{k}}-t^{\frac{\mu}{k}} \bigr\vert \biggl\vert f'\biggl(t \bigl(\lambda a+m(1-\lambda)r\bigr)+m(1-t) \biggl(\lambda r+(1-\lambda) \frac{a}{m}\biggr)\biggr) \biggr\vert \,\mathrm{d}t \\ &\quad\leq\biggl( \int^{1}_{0}1^{p}\,\mathrm{d}t \biggr)^{\frac{1}{p}} \biggl[ \int^{1}_{0} \bigl\vert (1-t)^{\frac{\mu}{k}}-t^{\frac{\mu}{k}} \bigr\vert ^{q} \\ &\qquad{} \times \biggl\vert f'\biggl(t\bigl(\lambda a+m(1-\lambda)r \bigr)+m(1-t) \biggl(\lambda r+(1-\lambda)\frac{a}{m}\biggr)\biggr) \biggr\vert ^{q}\,\mathrm{d}t \biggr]^{\frac{1}{q}} \\ &\quad=\biggl[ \int^{\frac{1}{2}}_{0} \bigl\vert (1-t)^{\frac{\mu}{k}}-t^{\frac{\mu }{k}} \bigr\vert ^{q} \biggl\vert f'\biggl(t\bigl(\lambda a+m(1-\lambda)r\bigr)+m(1-t) \biggl(\lambda r+(1-\lambda)\frac{a}{m}\biggr)\biggr) \biggr\vert ^{q} \,\mathrm{d}t \\ & \qquad{}+ \int^{1}_{\frac{1}{2}} \bigl\vert t^{\frac{\mu}{k}}-(1-t)^{\frac{\mu}{k}} \bigr\vert ^{q} \biggl\vert f'\biggl(t\bigl(\lambda a+m(1-\lambda)r\bigr)+m(1-t) \biggl(\lambda r+(1-\lambda)\frac{a}{m}\biggr)\biggr) \biggr\vert ^{q} \,\mathrm{d}t\biggr]^{\frac{1}{q}} \\ &\quad\leq\biggl\{ \int^{\frac{1}{2}}_{0}\bigl((1-t)^{\frac{\mu}{k}q}-t^{\frac {\mu}{k}q} \bigr) \\ & \qquad{}\times\biggl[t^{\alpha}\bigl\vert f'\bigl(\lambda a+m(1- \lambda)r\bigr) \bigr\vert ^{q}+m\bigl(1-t^{\alpha}\bigr) \biggl\vert f'\biggl(\lambda r+(1-\lambda)\frac{a}{m}\biggr) \biggr\vert ^{q}\,\mathrm{d}t\biggr] \\ & \qquad{}+ \int^{1}_{\frac{1}{2}}\bigl(t^{\frac{\mu}{k}q}-(1-t)^{\frac{\mu }{k}q} \bigr) \\ & \qquad{}\times\biggl[ t^{\alpha}\bigl\vert f'\bigl(\lambda a+m(1- \lambda)r\bigr) \bigr\vert ^{q}+m\bigl(1-t^{\alpha}\bigr) \biggl\vert f'\biggl(\lambda r+(1-\lambda)\frac{a}{m}\biggr) \biggr\vert ^{q}\biggr]\,\mathrm{d}t\biggr\} ^{\frac{1}{q}} \\ &\quad=\biggl\{ \biggl[\beta\biggl(\frac{1}{2},\alpha+1,\frac{\mu}{k}q+1 \biggr)-\beta\biggl(\frac{1}{2},\frac{\mu}{k}q+1,\alpha+1\biggr) \\ &\qquad{} +\frac{k}{\mu q+(\alpha+1)k}-\frac{k}{\mu q+(\alpha+1)k}\biggl(\frac {1}{2} \biggr)^{\frac{\mu q+\alpha k}{k}}\biggr] \bigl\vert f' \bigl(\lambda a+m(1-\lambda)r\bigr) \bigr\vert ^{q} \\ &\qquad{} -\biggl[\beta\biggl(\frac{1}{2},\alpha+1,\frac{\mu}{k}q+1\biggr)- \beta \biggl(\frac{1}{2},\frac{\mu}{k}q+1,\alpha+1\biggr)- \frac{k}{\mu q+(\alpha +1)k}\biggl(\frac{1}{2}\biggr)^{\frac{\mu q+\alpha k}{k}} \\ &\qquad{} +\frac{k}{\mu q+(\alpha+1)k}-\frac{2k}{\mu q+k}+\frac{2k}{\mu q+k}\biggl( \frac{1}{2}\biggr)^{\frac{\mu q}{k}}\biggr] m \biggl\vert f'\biggl(\lambda r+m(1-\lambda)\frac{a}{m}\biggr) \biggr\vert ^{q} \biggr\} ^{\frac{1}{q}}. \end{aligned}$$

Here, we use $(A-B)^{q}\leq A^{q}-B^{q}$ for any $A\geq B\geq0$ and $q\geq 1$. This ends the proof. □

### Theorem 4.3

*Let* $f:I\subseteq\mathbb{R}\rightarrow\mathbb{R}$ *be a differentiable mapping on* $I^{o}$ *along with* $a,r\in I$ *and* $0\leq a < mr$. *If* $\vert f' \vert ^{q}$ *for* $q>1$ *is* $(\alpha,m)$-*convex on* $[a,mr]$ *and* $f'\in L^{1}[a,mr]$, *then the following inequality for* *k*-*Riemann*-*Liouville fractional integral holds*:

4.3 $$\begin{aligned} &\bigl\vert \mathcal{T}_{k,\mu}(m,\lambda,r) \bigr\vert \\ &\quad\leq \biggl[\frac{2k}{\mu p+k}\biggl(1-\frac{1}{2^{\frac{\mu}{k}p}} \biggr) \biggr]^{\frac{1}{p}} \\ &\qquad{} \times\biggl[\frac{1}{\alpha+1} \bigl\vert f'\bigl(\lambda a+m(1-\lambda)r\bigr) \bigr\vert ^{q}+\frac{\alpha m}{\alpha+1} \biggl\vert f'\biggl(\lambda r+(1-\lambda)\frac {a}{m}\biggr) \biggr\vert ^{q}\biggr]^{\frac{1}{q}}, \end{aligned}$$

*where* $\lambda\in(0,1]\backslash\frac{1}{2}$, $k>0$, $\mu>0$ *and* $\frac{1}{p}+\frac{1}{q}=1$.

### Proof

Employing Lemma 2.1, Hölder’s inequality and the $(\alpha,m)$-convexity of $\vert f' \vert ^{q}$, we have

$$\begin{aligned} & \bigl\vert \mathcal{T}_{k,\mu}(m,\lambda,r) \bigr\vert \\ &\quad\leq\biggl[ \int^{1}_{0} \bigl\vert (1-t)^{\frac{\mu}{k}}-t^{\frac{\mu}{k}} \bigr\vert ^{p}\biggr]^{\frac{1}{p}} \\ &\qquad{} \times\biggl[ \int^{1}_{0} \biggl\vert f'\biggl(t \bigl(\lambda a+m(1-\lambda)r \bigr)+m(1-t) \biggl(\lambda r+(1-\lambda) \frac{a}{m}\biggr)\biggr) \biggr\vert ^{q}\,\mathrm {d}t \biggr]^{\frac{1}{q}} \\ &\quad\leq\biggl[ \int^{\frac{1}{2}}_{0}\bigl((1-t)^{\frac{\mu}{k}}-t^{\frac{\mu }{k}}\bigr)^{p}\,\mathrm{d}t+ \int^{1}_{\frac{1}{2}}\bigl(t^{\frac{\mu}{k}} -(1-t)^{\frac{\mu}{k}}\bigr)^{p}\,\mathrm{d}t \biggr]^{\frac{1}{p}} \\ & \qquad{}\times\biggl\{ \int^{1}_{0}\biggl[t^{\alpha}\bigl\vert f'\bigl(\lambda a+m(1-\lambda )r\bigr) \bigr\vert ^{q}+m\bigl(1-t^{\alpha}\bigr) \biggl\vert f' \biggl(\lambda r+(1-\lambda)\frac{a}{m} \biggr) \biggr\vert ^{q}\biggr]\,\mathrm{d}t\biggr\} ^{\frac{1}{q}} \\ &\quad\leq\biggl[ \int^{\frac{1}{2}}_{0}\bigl((1-t)^{\frac{\mu}{k}p}-t^{\frac {\mu}{k}p} \bigr)\,\mathrm{d}t+ \int^{1}_{\frac{1}{2}}\bigl(t^{\frac{\mu}{k}p} -(1-t)^{\frac{\mu}{k}p}\bigr)\,\mathrm{d}t\biggr]^{\frac{1}{p}} \\ &\qquad{} \times\biggl\{ \int^{1}_{0}\biggl[t^{\alpha}\bigl\vert f'\bigl(\lambda a+m(1-\lambda )r\bigr) \bigr\vert ^{q}+m\bigl(1-t^{\alpha}\bigr) \biggl\vert f' \biggl(\lambda r+(1-\lambda)\frac{a}{m} \biggr) \biggr\vert ^{q}\biggr]\,\mathrm{d}t\biggr\} ^{\frac{1}{q}}\\ &\quad=\biggl[\frac{2k}{\mu p+k}\biggl(1-\frac{1}{2^{\frac{\mu}{k}p}} \biggr) \biggr]^{\frac{1}{p}} \\ &\qquad{} \times\biggl[\frac{1}{\alpha+1} \bigl\vert f'\bigl(\lambda a+m(1-\lambda)r\bigr) \bigr\vert ^{q}+\frac{\alpha m}{\alpha+1} \biggl\vert f'\biggl(\lambda r+(1-\lambda)\frac {a}{m}\biggr) \biggr\vert ^{q}\biggr]^{\frac{1}{q}}, \end{aligned}$$

where we use the fact that $(A-B)^{q}\leq A^{q}-B^{q}$ for any $A\geq B\geq 0$ and $q\geq1$. This completes the proof. □

### Remark 4.1

If we take $\lambda=0$ or $\lambda=1$, we can deduce some new *k*-fractional integral trapezium-like inequalities from the results of Theorems 4.1, 4.2 and 4.3 and their related inequalities.
